# Supplementary material for: Vaccine-induced COVID-19 mimicry syndrome
Source: eLife. 2022 Jan 27;11:e74974. doi: 10.7554/eLife.74974 (PMC8846585; doi:10.7554/eLife.74974)

Blots shown in Figure 3 can be divided into 4 Blots for AD5.S (a-d), 4 blots for ChAdOx1-S (a-d)and 4 blots for AD26.COV2.S (a-d)


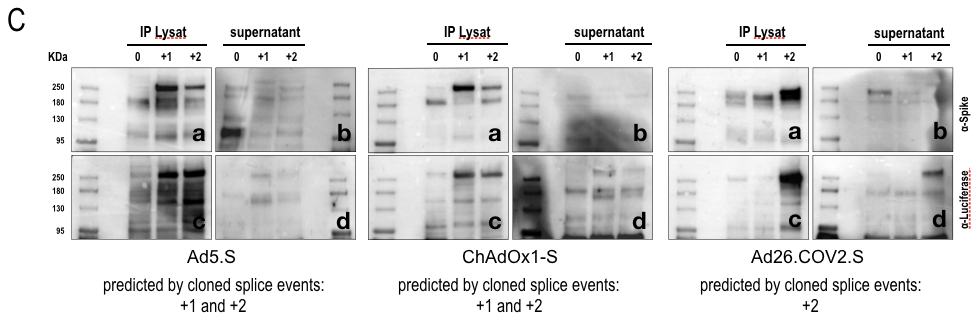


On subsequent pages 2-7, the original blots are shown from which these 12 blots of Figure 3 have been taken.

The right one of these 2 original blots have been used for Figure 3, Ad5.S c

This original blot are have been used for Figure 3, ChAdOx1 d and Ad26.COV2.S d

This original blot has been used for Figure 3, Ad5.S d

This original blots has been used for Figure 3, ChAdOx1-S a and Ad26.COV2.S a

The left of these 2 original blots have been used for Figure 3, Ad5.S a

The left blot, bottom, has been used for Figure 3, ChAd0x1-S b and the left blot, top, has been used for Ad26.COV2.s b

Agarose gels pictures shown in Figure 4 were retrieved from asingle agarose gel, where 4 different primer combinations were used to show splicing events


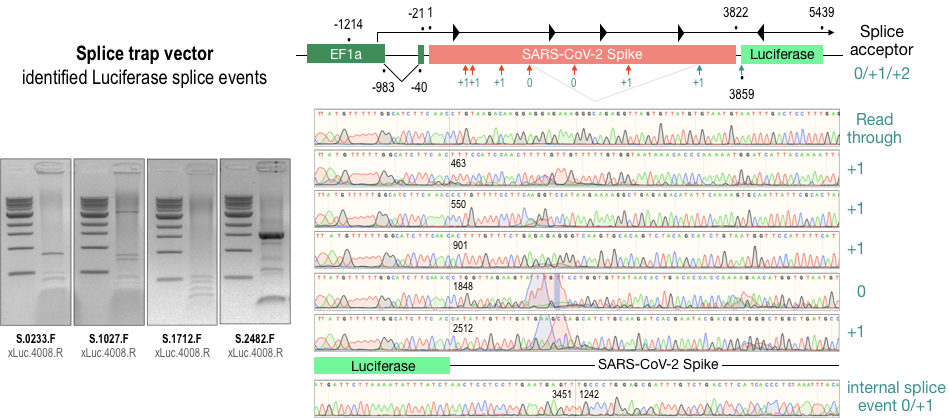


On subsequent page 9, the original gel picture is shown from which these 4 single pictures of Figure 4 have been taken.


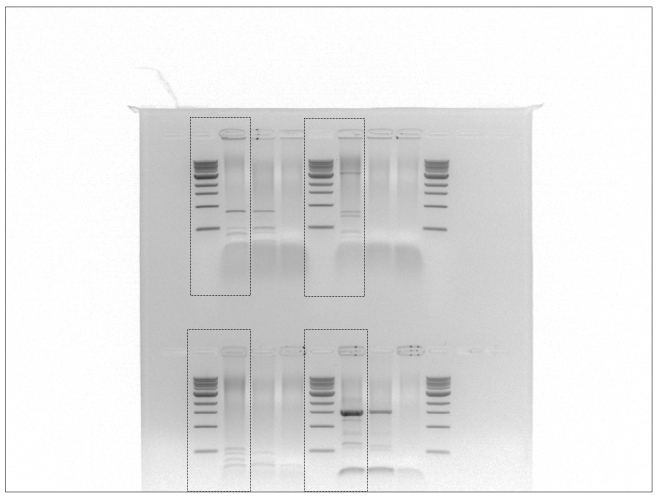


Agarose gels shown in Figure 5-7 were retrieved be divided into 4 gels for the different primer combinations


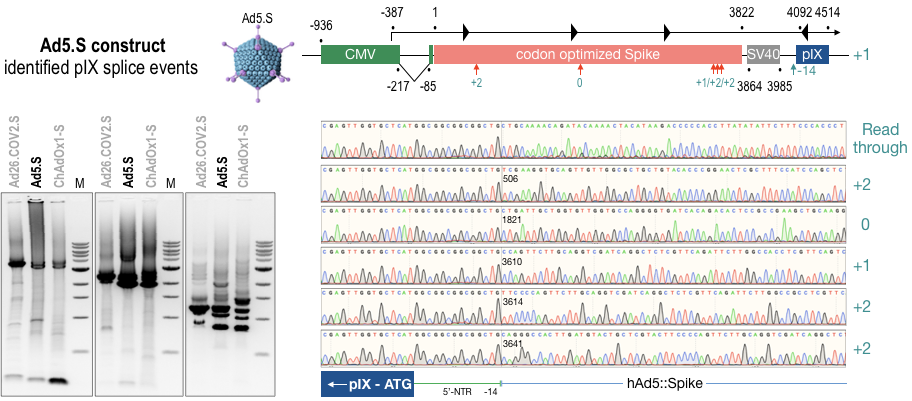


On subsequent page 11, the original gel picture is shown from which these 3 single pictures of Figure 5-7 have been taken.


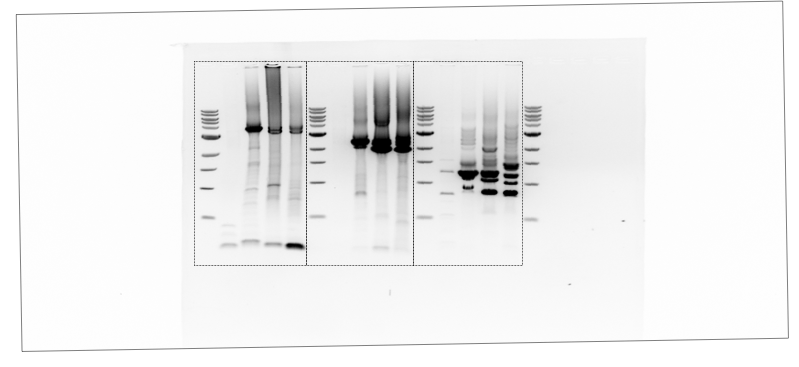


Agarose gels shown in Figure 9A-C were retrieved from 3 gels for the different primer combinations and cell lines


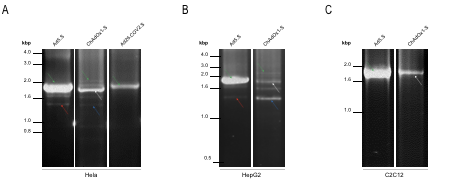


On subsequent pages 13-15, the original 3 gel pictures are shown from which these 7 single pictures of Figure 11A-C have been taken.

Figure 11A - Ad5.S, CaAdOx1.S and Ad26.COV2.S from Hela cells


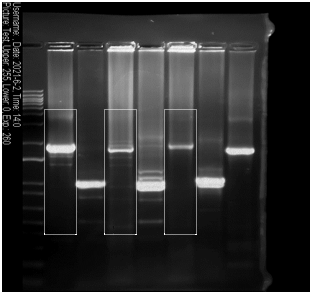


Figure 11B - Ad5.S and CaAdOx1.S from HepG2 cells


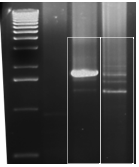


Figure 11C - Ad5.S, CaAdOx1.S and Ad26.COV2.S from C2C1 cells


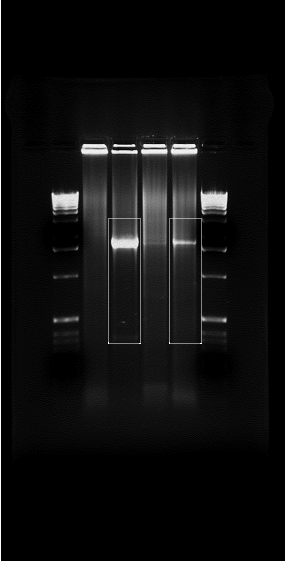

Supplement: Source data 1. [file elife-74974-data1.docx]
